# Materials and Applications of 3D Printing Technology in Dentistry: An Overview

**DOI:** 10.3390/dj12010001

**Published:** 2023-12-19

**Authors:** Min Jeong, Kyle Radomski, Diana Lopez, Jack T. Liu, Jason D. Lee, Sang J. Lee

**Affiliations:** 1 Department of Restorative Dentistry and Biomaterials Sciences, Harvard School of Dental Medicine, Boston, MA 02115, USA; min_jeong@hsdm.harvard.edu (M.J.); kradomski@hsdm.harvard.edu (K.R.); dianalopez@hsdm.harvard.edu (D.L.); jason_lee@hsdm.harvard.edu (J.D.L.); 2 Dexter Southfield, Brookline, MA 02445, USA; jacktl1688@gmail.com

**Keywords:** 3D printing, digital light processing, stereolithography, digital dentistry, dental material

## Abstract

Purpose. This narrative review aims to provide an overview of the mechanisms of 3D printing, the dental materials relevant to each mechanism, and the possible applications of these materials within different areas of dentistry. Methods. Subtopics within 3D printing technology in dentistry were identified and divided among five reviewers. Electronic searches of the Medline (PubMed) database were performed with the following search keywords: 3D printing, digital light processing, stereolithography, digital dentistry, dental materials, and a combination of the keywords. For this review, only studies or review papers investigating 3D printing technology for dental or medical applications were included. Due to the nature of this review, no formal evidence-based quality assessment was performed, and the search was limited to the English language without further restrictions. Results. A total of 64 articles were included. The significant applications, applied materials, limitations, and future directions of 3D printing technology were reviewed. Subtopics include the chronological evolution of 3D printing technology, the mechanisms of 3D printing technologies along with different printable materials with unique biomechanical properties, and the wide range of applications for 3D printing in dentistry. Conclusions: This review article gives an overview of the history and evolution of 3D printing technology, as well as its associated advantages and disadvantages. Current 3D printing technologies include stereolithography, digital light processing, fused deposition modeling, selective laser sintering/melting, photopolymer jetting, powder binder, and 3D laser bioprinting. The main categories of 3D printing materials are polymers, metals, and ceramics. Despite limitations in printing accuracy and quality, 3D printing technology is now able to offer us a wide variety of potential applications in different fields of dentistry, including prosthodontics, implantology, oral and maxillofacial, orthodontics, endodontics, and periodontics. Understanding the existing spectrum of 3D printing applications in dentistry will serve to further expand its use in the dental field. Three-dimensional printing technology has brought about a paradigm shift in the delivery of clinical care in medicine and dentistry. The clinical use of 3D printing has created versatile applications which streamline our digital workflow. Technological advancements have also paved the way for the integration of new dental materials into dentistry.

## 1. Introduction

Three-dimensional printing allows us to construct a three-dimensional object from a digital model. Compared with traditional subtractive manufacturing processes, 3D printing creates a complex shape or geometries that would be otherwise infeasible to construct by other technologies. With improvements in precision, accuracy, and 3D printable materials, various industries have been using 3D printing technology not only to create functional prototypes, but also to make a final product for their unique applications in an efficient and cost-effective manner. In medicine and dentistry, the evolution of 3D printing technology has also transformed the delivery of clinical care and provided various applications which improve the clinical outcome.

Technological advancements have allowed clinicians to provide more patient-specific medical devices. These devices are custom-made based on an individual patient’s anatomy, using imaging data and 3D models to create medical appliances [1]. There are two main types of manufacturing technology: subtractive and additive. Additive manufacturing technology, also known as 3D printing, provides versatile and customized applications beyond those possible through subtractive milling [2]. Researchers are exploring 3D bioprinting technology to replicate living tissues and organs for transplantation and to replace damaged tissues. Some of these printing applications have also spurred a paradigm shift in dental workflows. Among the technological advances that have played a role in this development are digital light processing (DLP) [3] and stereolithography (SLA) [1,3]. Other 3D printing technologies include fused deposition modeling (FDM), selective laser sintering (SLS), selective laser melting (SLM), photopolymer jetting, power binder printing, and laser bioprinting. However, due to various limitations, these 3D printing technologies have limited usage in dentistry.

**Figure 1:**
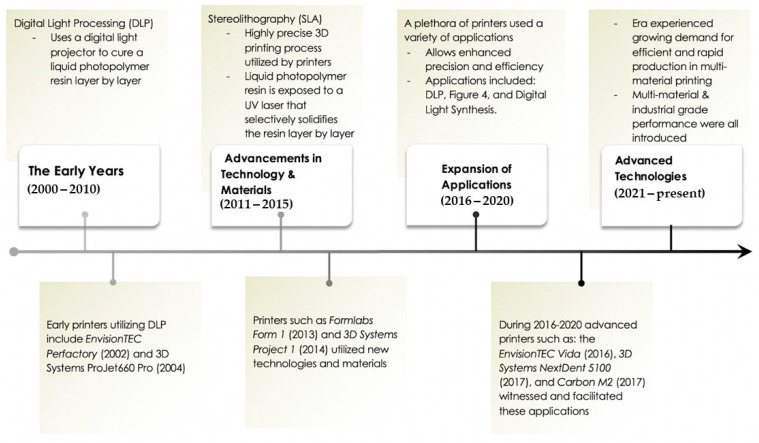
Historical timeline of 3D printing.

The most commonly used material in 3D printing technology is light-cured resin. Resin curing techniques have been the most influential in shaping the trajectory of practical dental 3D printing procedures [4]. The chronological development of curing techniques is divided into four time periods (Figure 1). “The Early Years (2000–2010)” are the beginning of digital light processing (DLP) technology [3]. This mechanism introduced the process of precision layer-by-layer printing [5,6]. The second phase, “Advancements in Materials and Technologies (2011–2015)”, utilized the stereolithography (SLA) technique, bringing high-resolution dental models into focus [7]. The following period, “Expansion of Applications (2016–2020),” was marked by the introduction of the DLP 3D printer, which expanded a wider range of dental applications [2].

The current era, “Advanced Technologies and Larger Platforms (2021—Present),” witnessed the unfolding of large-format dental 3D printing. This is designed for high-throughput production of dental models and appliances [5,8] and multi-material capabilities, providing dedication to dental- and medical-grade applications [8].

From previously established 3D printing mechanisms, we aim to chronicle the evolution of DLP and SLA technologies within the dental 3D printing landscape, highlighting their vital role in transforming not only dentistry but also the broader medical field [9,10]. These techniques have not only advanced in terms of precision and material diversity but have also opened new avenues for medical applications as a whole [1].

This paper will review the mechanisms of 3D printing technologies and how this technology has been applied in dentistry alongside the development of printable dental materials. Additionally, an overview of unique applications for 3D printing within different dental specialties will provide a holistic perspective on the impact of this technology in the dental field.

## 2. Methods

**Figure 2:**
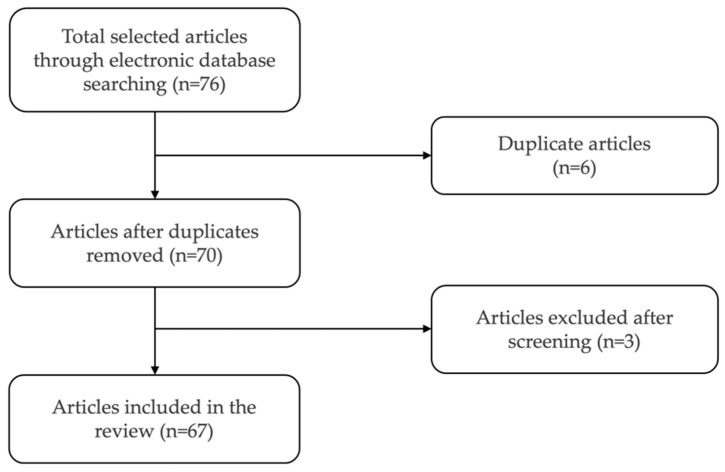
Flowchart of the article selection process.

Subtopics within 3D printing technology in dentistry were identified and divided among five reviewers. Electronic searches of the Medline (PubMed) database were performed with the following search keywords: “3D printing, digital light processing, stereolithography, digital dentistry, dental materials”, and combinations of the keywords. The reviewed articles were published between 1999 and 2023. For this review, only studies or review papers investigating 3D printing technology for dental or medical applications were included (Figure 2). Due to the nature of this review, no formal evidence-based quality assessment was performed, and the search was limited to the English language without further restrictions.

## 3. Three-Dimensional Printing Technologies

There are different mechanisms for how 3D printing technologies work. Depending on its 3D printing mechanism, the product has unique features which fit the use. Table 1 summarizes the advantages and disadvantages of various 3D print technologies.

### 3.1. Stereolithography (SLA)

**Figure 3:**
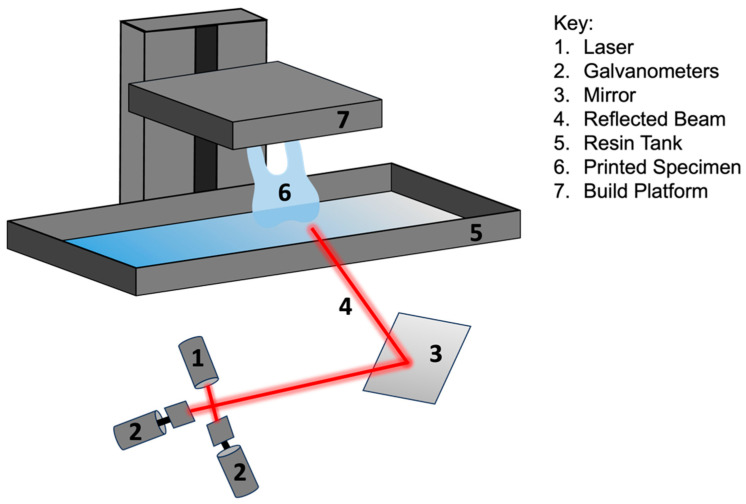
Diagram of stereolithography (SLA) technology.

Stereolithography (SLA) has proven to be a 3D printing technology with numerous applications, high speed, and high accuracy. SLA utilizes photochemical processes to cure liquid resins layer by layer, resulting in highly accurate and finely detailed designs [11] (Figure 3). The beam-curing capability of SLA technology is time-consuming but leads to highly accurate and smooth final products [11]. The technology’s ability to produce custom, patient-specific designs has developed significant interest within the dental community. As referenced in Table 1, SLA produces highly accurate designs and is often utilized in dentistry for temporary and permanent crown and fixed partial denture work, surgical guides, and templates as well as diagnostic casts and models [12]. One of the major downsides of the technology is that since the cured material follows a laser beam, the process can be time consuming, even for small designs [11]. SLA technology in dentistry has largely paved the way for a more efficient and less time-consuming approach to dental care.

### 3.2. Digital Light Processing (DLP)

**Figure 4:**
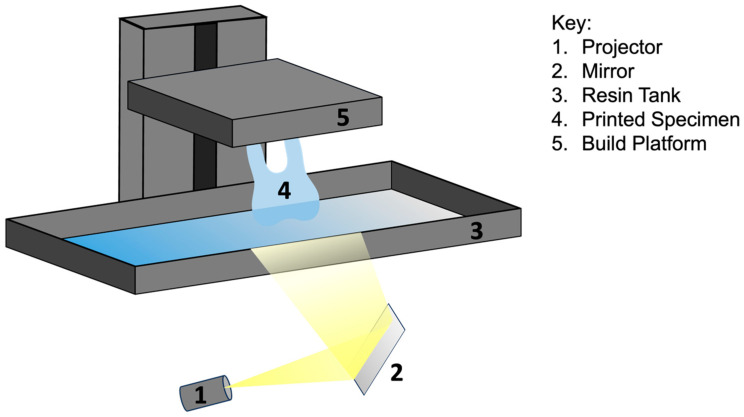
Diagram of digital light processing (DLP) technology.

Digital light processing (DLP) printing has emerged as an immensely valuable 3D printing technology, solving the issues of long fabrication durations [13]. DLP utilizes a light source to cure photopolymer resins layer by layer, resulting in highly precise and intricate designs [14] (Figure 4). This light-curing technology solves the issue of slower speeds seen in SLA printing, as DLP can cure an entire layer with one flash of light. The large disadvantage of this technology, though, exists around the size of each voxel. A voxel is essentially what a “pixel” is to resolution, except in a 3D perspective [15]. Therefore, a larger voxel size would lead to lower resolutions (blockier and squarer), while smaller voxels would lead to higher resolutions (smoother design) [15]. DLP printing currently produces clinically acceptable temporary and permanent restorations of crowns and fixed partial dentures as well as removable prosthetic devices [12]. Overall, DLP printing offers clinicians innovative time-saving solutions for more predictable treatment outcomes.

### 3.3. Fused Deposition Modeling (FMD)

Fused deposition modeling (FDM) is a useful printing modality with applications in many areas of healthcare. FDM utilizes thermoplastic filaments that are extruded once heated in a semi-solid state and deposited layer by layer [16]. The layers will harden when cooled yet will form a molecular bond with the heated filament as they are deposited onto the previous layer [16]. The technology provides great bonding of the material layers but only works with thermoplastic materials (Table 1). Currently, this technology has been used to produce occlusal appliances and has also been used in pharmaceutical applications, such as controlled-release drug delivery systems [12]. While fused deposition modeling provides useful applications throughout various healthcare settings, its use in dental applications is limited.

### 3.4. Selective Laser Sintering (SLS)

Selective laser sintering (SLS) has proven to be a highly time-saving 3D printing modality within the realm of prosthodontics. SLS utilizes a high-temperature laser to selectively fuse powdered materials [17]. The materials are wide-ranging, from ceramics to metals and even polymers [17]. This proves very useful as it is one of the only modalities that can produce high-density materials for dental applications [17]. The main disadvantage of this technology is that it requires a large infrastructure for proper printing [1]. SLS has demonstrated significant uses in dentistry in the fabrication of removable partial denture frameworks, which significantly reduces human error from traditional techniques [12]. Selective laser sintering provides a safer and more predictable outcome when compared to traditional casting of metal in dental applications. An alternative to SLS printing is selective laser melting (SLM). The SLM printing modality is comparable to SLS printing in terms of materials and processes, with the major difference being that in SLM, the material will be fully melted rather than sintered [18].

### 3.5. Photopolymer Jetting

Photopolymer jetting printing, commonly referred to as PolyJet 3D printing, provides a unique advantage to dentistry, namely the ability to print in multiple colors. PolyJet utilizes inkjet printheads to dispense droplets of fusing agent on multiple voxels of a powder bed [19]. This process will cause the polymer powder to melt and then be cured through infrared lights [19]. The technology’s ability to create multi-material and multi-color components yields huge advantages over other printing modalities. One of the major disadvantages revolves around the necessity of maintenance of the print heads, as they can clog easily [1]. Currently, PolyJet printing has displayed uses for the fabrication of dental models as well as temporary crowns [12]. The material does not currently provide great mechanical properties and for that reason possesses limited advantages in the oral environment [12]. Photopolymer jetting printing holds tremendous promise for revolutionizing the dental industry by providing multiple color options during a print, which is highly valued in esthetic dentistry.

### 3.6. Powder Binder Jetting

Powder binder printing, also known as binder jetting, is a useful modality for maxillofacial prostheses involving medical-grade silicones and biocompatible elastomers [20]. Binder jetting often employs a water-based binder to selectively bond layers of starch-based powder which is then infiltrated with silicone polymers [20]. The resulting material then undergoes post-processing to harden the material into acceptable properties [20]. The technology’s capability to produce patient-specific and color-matched maxillofacial designs is unmatched. These materials often have weaker mechanical properties and, as a result, are delicate [1]. As stated previously, powder binder jetting provides an extremely early and revolutionary solution to patient-specific maxillofacial applications [12]. Powder binder printing holds great promise in advancing dental manufacturing processes involving a less invasive production process for this unique patient population.

### 3.7. Laser Bioprinting (LAB)

Three-dimensional laser bioprinting (LAB) entered the market as an innovative amalgam of additive manufacturing and biotechnology. This modality has displayed excellent advancements for dental regenerative therapies. LAB printing utilizes precise laser-based techniques, which enable the layer-by-layer deposition of bioinks, including living cells and various types of biomaterials [21]. This innovative technology developed printing in the field of producing tissue-engineered constructs for periodontal regeneration, bone augmentation, and oral mucosal reconstruction [21]. Overall, 3D laser bioprinting holds remarkable promise in reshaping the landscape of dental therapies, offering clinicians innovative tools to engineer patient-specific, regenerative solutions for complex oral and maxillofacial challenges with the potential to enhance patient well-being and quality of life.

## 4. Dental Materials in 3D Print Technology

Printable dental materials are rapidly advancing, with research focused on the development of additive manufacturing (AM) printing parameters to fine-tune the mechanical properties of conventionally used materials. In addition, biocompatibility is important for 3D printing materials used in dentistry. Compared to conventional dental resins, 3D printing resins have been proven to have similar biocompatibility [22,23,24]. This biocompatibility can be further improved with post-processing like curing and washing [24]. In Table 2, a summary is provided of some common 3D-printed materials and their measured properties compared to their comparable conventional counterparts. The main printable materials can be categorized into synthetic polymers, metals, and ceramics.

### 4.1. Synthetic Polymers

Polymers are the most common materials used for dental applications due to the low cost and diverse properties and capabilities. Examples of polymers include polyether ether ketone (PEEK), polycaprolactone (PCL), polymethyl methacrylate (PMMA), polylactic acid (PLA), poly (lactic-co-glycolic acid) (PLGA), and ultraviolet (UV) resins. In a systematic review of in vitro studies, Valenti et al. [25] found that the mechanical properties of AM-printed polymeric materials were generally lower than those of materials produced by conventional methods. Wesemann et al. [26] investigated the wear resistance and mechanical properties of AM-printed occlusal appliances compared to the conventional injection molding method and found that there was a significant difference between their mechanical properties. Prpic’ et al. [27] investigated the mechanical properties of AM-printed PMMA compared to conventional heat-polymerized and injection pressing PMMA, as used for denture bases. The authors found that although the AM-printed group had the lowest flexural strength, it still met the ISO requirement of 65 MPa.

### 4.2. Metals

The mechanical properties of titanium (Ti) and cobalt-chromium (Co-Cr) alloys are ideal for many dental applications. A review by Revilla-Leon et al. [28] compared different printed alloys with conventional casting methods. The authors found that while their mechanical properties were satisfactory, improvements in 3D-printed metals and ceramic interfaces are required to match the precision obtained with conventional casting methods. Previous studies have noted higher hardness values for 3D-printed CoCr metal alloys (371 ± 10 HV) compared to conventional casting methods [29]. In general, an increase in fit accuracy is reported in AM-printed removable partial denture metal clasps, compared to conventional casting methods [25].

### 4.3. Ceramics

Ceramic materials are considered a favorable material for dental restorations due to their excellent mechanical properties, biocompatibility, good abrasion and corrosion resistance, and esthetic properties. This category can be further divided into glass, zirconia, and alumina ceramics. A challenge with 3D printing ceramics is inherent to their high melting points and introduction of cracks during the cooling processes. Additionally, the characteristics of the raw materials affect their porosity and final mechanical properties. For AM-printed ceramics, reports of their mechanical properties are inconsistent [25]. This is largely due to the different types of ceramic materials, variability in prosthetic use, and printing parameters [4]. This category of dental materials is a prime example of an area where further laboratory and clinical investigation is required to achieve widespread use.

**Table 2:** Summary of mechanical properties for oral tissue, common AM-printed materials, and conventional dental materials.

Materials	Density (g/cm^3^)	Martens Hardness (N/mm^2^)	Vickers Hardness (GPa)	Tensile Strength (MPa)	Elastic Modulus (GPa)	Bending Strength (MPa)
Cortical bone * [30]	1.92	NP	NP	104–121	6–30	225
Dentin * [30]	NP	468.2 ± 30.8	NP	104	12–18.6	NP
Dental enamel [30]	NP	2263.6 ± 405.2	NP	47.5	40–83	NP
PEEK * [30]	1.3	189.55 ± 16.89	NP	87.53–100	3–4	99.25–170
PEEK, FDM [30]	NP	NP	NP	97.34	2.6–3.45	104.65
PMMA * [26,30]	1.18	180	19.9 ± 1.0	NP	2.3 ± 0.3	85 ± 16
PMMA, SLA [26]	NP	NP	18.1 ± 1.0	NP	1.2 ± 0.3	95 ± 9
PMMA, DLP [26]	NP	NP	14.7 ± 1.5	NP	0.7 ± 0.2	37 ± 6
PLA * [31]	1.25	NP	NP	59	3500	106
PLA, FDM [31]	NP	NP	NP	28–48	2000	NP
Ti * [30]	4.5	300–400	NP	954–976	102–110	NP
Ti, SLS [28]	4.42	NP	38	1089	129	NP
CoCr * [30]	6.5	1200	350	680	205	800–1400
CoCr, SLS [28]	8.3	NP	350–450	1100	200	NP
ZrO_2_ * [32]	NP	5000–15,000	NP	115–711	100–250	177–1000
ZrO2, SLA [32]	5.97	NP	12.6	NP	209.4	300–1000
Al_2_O_3_ * [32]	NP	22,000	NP	267	380	500
Al_2_O_3_, SLA [32]	NP	NP	NP	NP	NP	271.7–273.8

*: conventional materials. PEEK: Polyether ether ketone. PMMA: Polymethyl methacrylate. PLA: Polylactic acid. Ti: Titanium. CoCr: Cobalt-chromium. ZrO_2_: Zirconium dioxide. Al_2_O_3_: Aluminum oxide. FDM: Fused deposition modeling. SLA: Stereolithography. SLS: Selective laser sintering. NP: Not provided.

## 5. Applications of 3D Printing in Dentistry

Three-dimensional printing has brought new applications along with the advancement of dental materials. Its versatile applications have provided more predictable clinical outcomes with precision and accuracy in many dental specialties. Table 3 summarizes the unique applications of 3D printing in various dental specialties.

### 5.1. Prosthodontics

#### 5.1.1. Crown and Fixed Partial Dentures (FPD)

Three-dimensional printing has always been a technology of great interest in the field of prosthodontics. This has become more apparent recently, with 3D printing technologies, such as SLA and DLP, becoming more widely utilized to fabricate provisional or definitive crowns and FPDs (Table 3). Precise virtual models are prepared by scanning the prepared teeth and implant scan bodies intraorally. Computer-aided design (CAD) software program (3Shape Dental System 2022, 3Shape A/S, Copenhagen, Denmark) is used to design the prostheses on these virtual models. Then, the designed prostheses are printed using a 3D printer. Compared to traditional methods or milling technology for fabricating crowns and FPDs, a low-cost 3D printer can fabricate precise restorations using fewer materials in a shorter production time [10,33,34]. Some studies have even reported that provisional crowns fabricated using 3D printing are more accurate (better edges and better internal fit) than the ones fabricated using traditional or milling methods [35,36,37].

#### 5.1.2. Complete and Removable Partial Dentures (RPD)

Although there have been significant improvements in denture material and techniques, the denture fabrication process can be challenging in patients with a severe gag reflex, tumor resection, temporomandibular joint disease, or oral deformities [38,39]. With the advent of intraoral scanning technology and 3D printing, denture fabrication has become a patient-friendly procedure with a shorter production time [40]. It also reduces traditional laboratory steps, which can lead to fewer inherent errors and better adaptability [41,42]. The traditional process of waxing and investing RPD frameworks is time-consuming, technique-sensitive, and cumbersome. Wax and cast distortion from this traditional process can lead to poor fit of framework, pressure-induced mucosal lesions, and ridge resorption [43]. A recent in vitro study showed that RPD frameworks fabricated by SLM printing resulted in a better fit than traditional lost-wax and metal casting techniques [44]. Another author also reported that 3D-printed frameworks provide more uniform contact pressure, which can reduce the risk of residual ridge resorption [10].

### 5.2. Implantology

The application of 3D printing technology in implantology serves to optimize and simplify surgical procedures with higher accuracy and predictability, thereby reducing surgical risks and improving efficiency. Traditional surgical guides are usually designed based on two-dimensional panoramic radiographs, which often lead to inaccuracy due to distortion and insufficient resolution of the radiographic image. With the introduction of advanced digital technology, surgical guides are now designed using CAD software based on cone beam computed tomography (CBCT) and intraoral scans, then printed using a 3D printer. With this technology, the accuracy of these 3D-printed surgical guides has significantly improved. Tahmaseb et al. compared and found a significant difference between the accuracy of traditional surgical guides and 3D-printed surgical guides that were printed by SLA printers [45]. For traditional surgical guides, the average distance deviations at the entrance and at the vertex were 2.1 mm and 1.5 mm, respectively [45]. Meanwhile, the average distance deviations at the entrance and at the vertex of the 3D-printed surgical guide were 0.9 and 1.0 mm, respectively [45]. Other advantages of 3D printing surgical guides over traditional ones are lower investment cost, shorter surgical time, simpler surgical process, and better adaptation to the patient’s geometry [46,47].

### 5.3. Oral and Maxillofacial

#### 5.3.1. Surgical Guides and Templates

Additive manufacturing technology has been used for three decades in the oral and maxillofacial field of dentistry for model fabrication, diagnosis, surgical planning, surgical guide and template fabrication, and custom implant manufacturing [48,49]. Similar to 3D-printed surgical guides for implant surgery, surgical guides and templates are designed based on the obtained CT image and CAD software analysis of the maxillomandibular defect. A 3D-printed guide in combination with a 3D-printed patient-specific titanium template provides stability during the operation and ensures the precise placement of bone segments [50]. In addition, these guides and templates result in fewer defects, higher accuracy, better margin control, and bone compromises [48].

#### 5.3.2. Custom Implants

The capability of 3D printing technology to design and print complex geometries has been used to fabricate custom dental implants. Three-dimensional printers, such as SLS and SLM, have the ability to print in titanium or in implantable polymer, particularly polyether ether ketone, to fabricate dental implants with adjustable porosity and mechanical properties [49].

However, 3D printing technology is still often used in conjunction with conventional pressing and milling technologies to fabricate implants, because pressing and milling have their advantages as well, such as reduced post-processing, fast production, and predictable use of uniform and homogenous materials [1].

#### 5.3.3. Maxillofacial Prostheses

Often, maxillofacial defects are complex in shape and size, and 3D printing technology can be extremely beneficial in fabricating prostheses for these defects due to its ability to print complex geometrics. A combination of scanning technology and 3D printing is more comfortable for patients and provides a prosthesis with higher accuracy and better fit to the defect area [48,51]. Other benefits of implementing 3D printing technology include reduced manufacturing time, decreased number of appointments, and repeatability allowing multiple prostheses [52].

### 5.4. Orthodontics

Nowadays, in orthodontics, 3D printing technology is primarily used to fabricate orthodontic aligners for treating malocclusion. Removable, clear aligners are an alternative to conventional orthodontic braces, with improved oral hygiene and esthetics [53].

Previously, 3D printing technologies such as SLA or FDM were used only for printing models, and aligners were produced by a thermoforming process using thermoplastic materials [53]. However, the thermoforming procedure itself as well as the intraoral environment can alter the properties of the material, which eventually affect its overall performance [54].

More recently, direct 3D-printed aligners have become more popular, offering a better fit, higher efficacy, and reproducibility, without altering material properties [55]. Tartaglia et al. reported that the use of direct 3D-printed aligners is a more stable way to align the teeth than thermoformed manufactured aligners due to higher accuracy, higher load resistance, and lower deformation [55].

Three-dimensional printing technology is also applied in gnathology for management of temporomandibular joint disorders (TMDs). The therapeutic position is digitally planned based on kinematic tracing record, and customized intraoral appliances can be digitally designed and printed using a 3D printer to increase the accuracy of the therapeutic position [56].

### 5.5. Endodontics

Three-dimensional printing technology has been applied to every field of dentistry, and endodontics is not an exception. In endodontics, 3D printing technology has served various purposes, including access cavity preparation, apicoectomy, autotransplantation, education, and training [57]. There are many studies reporting the high accuracy of guided cavity preparation using a 3D-printed access guide [58,59,60]. Buchgreitz et al. reported the mean deviation of access cavities lower than 0.7 mm [58]. Similarly, Zehnder et al. and Connert et al. reported small deviations of 0.12–0.34 mm from the intended access and a mean angular deviation of less than two degrees [59,60].

Guided apicoectomy, endodontic microsurgery (EMS), requires a 3D-printed surgical guide to perform targeted osteotomy and root resection. As in other specialties, the surgical guide is designed and printed based on CBCT and CAD software. This application of 3D printing technology results in higher accuracy of osteotomies than the traditional free-hand technique [57,61,62]. In addition, 3D-printed guides for apicoectomy allow for easier inspection of root apices, smaller osteotomies, lower risk of nerve or sinus perforation, better root-end preparation, better healing, and shorter surgical time [57,61,62].

### 5.6. Periodontics

#### 5.6.1. Scaffolds for Hard and Soft Tissue Regeneration

The application of 3D printing in periodontics is useful for both hard and soft tissue regeneration as well as guided gingivectomy. There has been a lot of research focusing on the fabrication of 3D-printed scaffolds for hard and soft tissue regeneration. The concept of additive biomanufacturing using 3D printing technology serves to restore the resorbed periodontal tissue and bone deficiencies in a customized manner [48].

Three-dimensional printing enables the custom printing of scaffolds that can be loaded with stem cells, where the stem cells can be placed at precise locations, allowing more intimate contact with bone surfaces [63]. These advantages can lead to a better healing process and better esthetic results than conventional scaffolds [48]. Three-dimensional printing can also be used for soft tissue regeneration. Recently, 3D-printed soft tissue grafts have been developed for keratinized tissue augmentation. These printed soft tissue grafts can cover larger and more complicated defects with high accuracy, without being limited by donor site availability [64].

#### 5.6.2. Gingivectomy Surgical Guide

A common application of 3D printing in periodontics is the use of a surgical guide for gingivectomy and smile designing [48]. With the help of intraoral scanning and CAD software, a patient-specific surgical guide for esthetic gingivectomy can be designed and printed. Using the surgical guide, more esthetic results can be achieved due to their accuracy, precision, and customization [48].

## 6. Future Directions

The use of 3D printing in the dental field is rapidly on the rise. Due to the improved accuracy, superior efficiency, and growing accessibility of the technology, 3D printing is quickly becoming the favored method of computer-aided manufacturing in the field. As printing technology and printable materials continue to advance, we can envision a future where prostheses and even biomaterials can be infinitely customized to fit the needs of patients. Currently, there are efforts to incorporate 4D printing into the dental field. With 4D printing technology, 3D-printed materials have the capacity to alter their shape over time in respond to external stimuli such as pressure, heat, light, or humidity to change form or physical properties even after the printing process is complete [63]. Potential applications could include printed biomaterials that expand to intimately fit the contours of a complex hard tissue defect, or a restoration that expands and contracts in perfect unison with the surrounding tooth structure in response to temperatures. In endodontics, 4D printing technology may be used to produce instruments with the ability of shape memory to adapt to canal curvature to prevent instrument separation. In addition, 4D printing technology may be utilized in the fabrication of dental implants by altering the hardness of the apical part to prevent nerve damage and sinus perforation [65]. Another promising printing technology includes computed axial lithography, which can create the entire object at once by having light polymerization applied at many directions [12]. This technology has the capacity to produce objects at a faster production rate and with low-viscosity materials [12]. Depending on the directions in which the technology and materials evolve, 3D printing will continue to play a significant part in the dental field.

## 7. Conclusions

This review article gives an overview of the history and evolution of 3D printing technology as well as its associated advantages and disadvantages. Current 3D printing technologies include stereolithography, digital light processing, fused deposition modeling, selective laser sintering/melting, photopolymer jetting, powder binder, and 3D laser bioprinting. The main categories of 3D printing materials are polymers, metals, and ceramics. Material selection and applications in dentistry have expanded significantly over the years alongside the ever-evolving 3D printing technologies. Although there are some limitations in printing accuracy and quality, 3D printing technology is now able to offer us a wide variety of potential applications in different fields of dentistry, including prosthodontics, implantology, oral and maxillofacial, orthodontics, endodontics, and periodontics. With further research being conducted, 3D printing will continue to elevate the quality and delivery of patient care, education, and research in the years to come.

## Figures and Tables

**Table 1:** Advantages and disadvantages of each 3D printing technology.

3D Technology	Advantages	Disadvantages
Stereolithography (SLA)	Quick production speed; Precise and highly accurate; Can accommodate complex designs; Numerous material options.	Production can be slower compared to other printers; High post-processing requirements.
Digital light processing (DLP)	High speed; Precise and highly accurate; Can accommodate complex designs; Numerous material options.	Arguably lower quality than other printers; Limited by voxel size.
Fused deposition modeling (FDM)	Cheaper technology; Great layer bonding.	Only thermoplastic materials.
Selective laser sintering (SLS) and selective laser melting (SLM)	Can print polymers or metals; Batch production; No supports needed.	Requires high printing infrastructure; Use of fine powders can he hazardous.
Photopolymer jetting	Extremely high resolution; Can print with multiple colors on one single print.	Low mechanical properties; Limited heat resistance; Costly maintenance of printer heads.
Powder binder printing	Wide range of unique materials; High speed printing.	Low mechanical properties; Low resolution; High waste of material.
3D laser bioprinting (LAB)	Only option to print living cells and other biomaterials; Completely unique.	Costly; Very specific conditions to produce viable biomaterials.

**Table 3:** Applications of 3D printing.

Specialty	Applications	Technology	Advantages
Prosthodontics	Crowns and fixed partial dentures	SLA, DLP, photopolymer jetting	Low costs; Reducing time consumption; Good adaptability; Precise.
	Complete dentures	SLA, DLP	Patient-friendly; Fewer laboratory steps; High accuracy; Close adaptability.
	Removable partial dentures	SLS, SLM, EBM	Reducing manufacturing time and cost; Minimizing operation errors; High accuracy; Good marginal fit; Good mechanical properties.
Implantology	Surgical guide	SLA, DLP, photopolymer jetting	Reducing operation errors; Simple operation; Accurate.
	Custom tray	SLA, FDM	High efficiency; High accuracy.
Oral and maxillofacial	Surgical guide and template	SLA, DLP, photopolymer jetting	High accuracy; Reducing operation time; Minimizing operation errors/injuries.
	Custom implants	SLS, photopolymer jetting	High mechanical properties; Adjustable porosity.
	Maxillofacial prostheses	SLA, photopolymer jetting, SLS, FDM	Patient comfort; Precise; Higher accuracy and better fit.
Orthodontics	Aligners	SLA, DLP, FDM	Accurate; Predictable; Reducing production time; Saving money.
	Orthotic appliances	SLA	Good mechanical properties.
Endodontics	Surgical guide	SLA, photopolymer jetting	Improving accuracy; Less invasive; Saving time; Reducing operation errors/injuries.
Periodontics	Scaffolds for hard and soft tissue regeneration	LAB	Precise; More intimate contact of scaffolds with bone surfaces; Larger quantity; Permitting more complicated designs; Accurate; Reducing post-operation discomfort.
	Gingivectomy surgical guide	SLA, DLP, photopolymer jetting	Accurate; Customized; Precise.

SLA: Stereolithography. DLP: Digital light processing. SLS: Selective laser sintering. SLM: Selective laser melting. EBM: Electron-beam additive manufacturing. FDM: Fused deposition modeling. LAB: Laser-assisted bioprinting.

## Data Availability

Not applicable.
